# The effect of coumaryl alcohol incorporation on the structure and composition of lignin dehydrogenation polymers

**DOI:** 10.1186/s13068-017-0962-2

**Published:** 2017-11-30

**Authors:** Anne E. Harman-Ware, Renee M. Happs, Brian H. Davison, Mark F. Davis

**Affiliations:** 1 Biosciences Center, National Renewable Energy Laboratory, 15013 Denver West Parkway, Golden, CO 80401 USA; 2 Bioenergy Science Center, National Renewable Energy Laboratory, 15013 Denver West Parkway, Golden, CO 80401 USA; 3 Bioenergy Science Center, Oak Ridge National Laboratory, Oak Ridge, TN 37831 USA

**Keywords:** Lignin, Dehydrogenation polymer, Coumaryl alcohol, Biomass recalcitrance

## Abstract

**Background:**

Lignin dehydrogenation polymers (DHPs) are polymers generated from phenolic precursors for the purpose of studying lignin structure and polymerization processes

**Methods:**

Here, DHPs were synthesized using a Zutropfverfahren method with horseradish peroxidase and three lignin monomers, sinapyl (S), coumaryl (H), and coniferyl (G) alcohols, in the presence of hydrogen peroxide. The H monomer was reacted with G and a 1:1 molar mixture of S:G monomers at H molar compositions of 0, 5, 10, and 20 mol% to study how the presence of the H monomer affected the structure and composition of the recovered polymers.

**Results:**

At low H concentrations, solid-state NMR spectra suggest that the H and G monomers interact to form G:H polymers that have a lower average molecular weight than the solely G-based polymer or the G:H polymer produced at higher H concentrations. Solid-state NMR and pyrolysis–MBMS analyses suggest that at higher H concentrations, the H monomer primarily self-polymerizes to produce clusters of H-based polymer that are segregated from clusters of G- or S:G-based polymers. Thioacidolysis generally showed higher recoveries of thioethylated products from S:G or S:G:H polymers made with higher H content, indicating an increase in the linear ether linkages.

**Conclusions:**

Overall, the experimental results support theoretical predictions for the reactivity and structural influences of the H monomer on the formation of lignin-like polymers.

**Electronic supplementary material:**

The online version of this article (10.1186/s13068-017-0962-2) contains supplementary material, which is available to authorized users.

## Background

Lignin in plant cell walls has been shown to contribute to biomass recalcitrance complicating the thermochemical and biological processes used to convert biomass to chemicals and fuels [1–5]. Efforts have been made to reduce biomass recalcitrance by decreasing the total amount of lignin present, by changing the ratio of monomers and by modifying the types of linkages that occur in the lignin polymers [6, 7]. The incorporation of the coumaryl (H) monomer in lignin in biomass could result in a decrease in the molecular weight of lignin and subsequently lead to reduced biomass recalcitrance [4]. Additionally, computational studies have indicated that the incorporation of the H monomer into lignin polymers results in the formation of dilignol compounds that do not support chain elongation [8]. The H monomer has been implicated to act as a “capping” agent that stops the polymerization of lignin and results in lower molecular weight polymers. Quantum mechanical calculations have determined the H monomer to be more reactive than the other two common lignin monomers [sinapyl (S) and coniferyl (G) alcohols] for self- and cross-coupling during dimerization, which has implications on the types of linkages formed in the presence of H monomers [9]. Observations have shown that biomass containing high-H monomer content in the lignin may contain mostly H lignin-like polymers that separate from the rest of the lignin S:G framework [4].

**Fig. 1:**
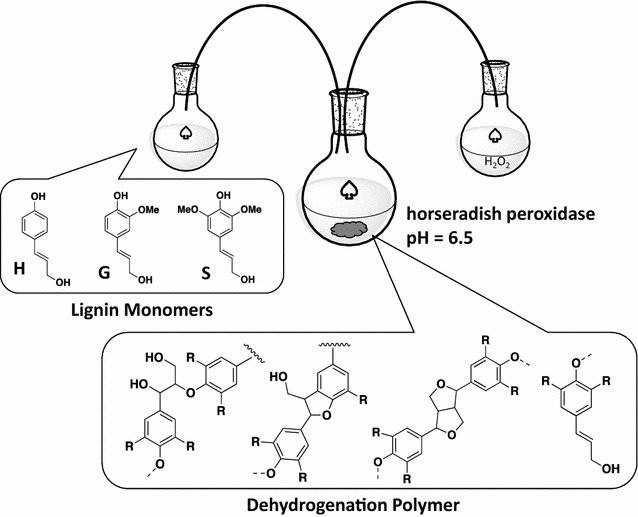
Zutropfverfahren synthesis of lignin dehydrogenation polymers

Lignin dehydrogenation polymers (DHPs) are phenylpropanoid polymers synthesized in the presence of hydrogen peroxide and an enzyme such as horseradish peroxidase, mimicking the proposed free radical condensation that occurs during lignification in vivo [10]. Lignin DHPs have been synthesized with and without various parameters to approximate cell wall conditions (incorporating redox shuttles, changing pH, etc.) to produce polymers that appear structurally similar to native lignins [11–13]. Without incorporating cell wall conditions and other influences such as redox shuttles and nucleophilic reagents, DHPs synthesized using bulk polymerization and Zutropfverfahren (slow, drop-wise, schematic shown in Fig. 1) methods typically have higher abundances of β–β bonds and fewer β-*O*-4 bonds than natural (native and extracted) lignins [10, 11, 13–15]. Hence, simple lignin DHP systems, which reflect only the thermodynamic tendencies of the monomers to polymerize with minimal influence of other species (besides aqueous or a buffered environment), can be used to understand the reactivity of the lignin monomers.

The purpose of this work was to synthesize lignin dehydrogenation polymers with varying amounts of the coumaryl (H) monomer in the presence of coniferyl (G) and sinapyl (S) monomers. While one study characterized the effects of H monomers on S- and G-based polymers by pyrolysis–GC/MS [16], thorough characterization of lignin DHPs incorporating varying amounts of H monomers has not been established. Based on density functional theory (DFT) studies, we hypothesize that there will be significant changes in the structure of G and S:G (50:50) polymers when synthesized in the presence of H monomers. Differences in lignin DHP structures incorporating varying amounts of H monomers could then be attributed to intrinsic thermodynamic differences in the reactivity of the monomers and polymers (separate from any biochemical control in biomass cell walls or cell wall conditions). Additionally, we hypothesized that H would preferentially self-couple as predicted in density functional theory (DFT) calculations and as observed in low-lignin alfalfa [4, 8, 9].

## Methods

### Lignin dehydrogenation polymer synthesis

Coniferyl alcohol (98%) and sinapyl alcohol (98%, GC) were obtained from Sigma Aldrich and p-coumaryl alcohol was purchased from BOC Sciences. Approximately 100 mg of monomers or monomer mixture was added to 100 mL pH 6.5 potassium phosphate buffer with the addition of 1 mL of ethanol (HPLC grade). Coniferyl alcohol (G monomer) and/or sinapyl alcohol (S monomer) were combined with coumaryl alcohol (H monomer) in 100 mL pH 6.5 potassium phosphate buffer with the addition of 1 mL of ethanol (200 proof) (the exact masses depended on the molar ratios; in each case, the monomers were mixed to generate a total of approximately 100 mg of monomer mixture). Polymers synthesized included H, S, G, G with 0–20% (molar) H, and S:G molar ratios of 1 with 0–20% (molar) H. Polymers are described as their molar ratios to indicate the degree of which H has been incorporated. For example, S:G:H (47.5:47.5:5) is S:G = 1 with 5 mol% H and the remaining 95% being equal molar S and G monomers. 30% hydrogen peroxide was diluted in water to make 100 mL of 0.04% hydrogen peroxide and 5 mL of this was added to a reaction flask containing 25 mL of potassium phosphate buffer. 1 mg of horseradish peroxidase (Sigma Aldrich, 250–330 units/mg) was added to the flask. Both the remaining hydrogen peroxide solution and the monomer solution in phosphate buffer were added separately using peristaltic pumps at 2.4 mL/h each at room temperature while stirring until all the solutions were dispensed in the reaction flask. After 24 h, an additional 1 mg of horseradish peroxidase was added to the reaction flask. The reaction was allowed to continue for 30 h after the entire solutions were dispensed for a total reaction time of approximately 72 h. The reaction solution was centrifuged for 4 h and the precipitate was washed three times with 15 mL of water (45 mL total) and centrifuged (4300×*g*) for an additional 1 h.

### Gel permeation chromatography

The resulting lignin dehydrogenation polymers were acetylated using acetic anhydride. DHP lignin (10 mg) was acetylated in a mixture of pyridine (0.5 mL) and acetic anhydride (0.5 mL) at 40 °C for 24 h with stirring. The reaction was terminated by addition of methanol (1.0 mL increments). The acetylation solvents were then evaporated from the samples at ambient temperature under a stream of nitrogen gas. Addition of methanol and evaporation were continued until all solvents were removed. The samples were further dried in a vacuum oven at 40 °C overnight. The dried, acetylated DHPs were dissolved in tetrahydrofuran (THF, Baker HPLC grade) and filtered (0.45-µm nylon membrane syringe filters) before analysis. GPC analysis was performed using an Agilent 1050 HPLC with three GPC columns (Polymer Laboratories, 300 × 7.5 mm) packed with polystyrene-divinyl benzene copolymer gel (10-µm beads) having nominal pore diameters of 10^4^, 10^3^, and 50 Å using a diode array detector measuring absorbance at 260 nm (band width 40 nm). The eluent, THF, had a flow rate of 1.0 mL/min. An injection volume of 20 µL was used. Polystyrene standards (Agilent Technologies) were used to calibrate for apparent weight average molecular weight (*M*_*w*_).

### Thioacidolysis of lignin dehydrogenation polymers

Thioacidolysis analysis of the polymers was performed according to the method reported by Harman-Ware et al. [17] to analyze thioethylated products from ether-linked (β-*O*-4 structures) monomers. Briefly, the thioacidolysis reagent consisted of 2.5% boron trifluoride diethyl etherate (> 47.5% BF_3_, Sigma Aldrich), 10% ethanethiol (97%, Alfa Aesar), and 87.5% dioxane by volume and contained bisphenol-E internal standard (reagent grade, TCI Chemical) at a concentration of 0.05 mg/mL. 1000 μL of the thioacidolysis reagent was added to the vial containing 1 mg of lignin DHP polymer, purged with nitrogen, capped, and heated to 100 °C for 4 h. After the reaction was neutralized and acidified, the products were extracted using ethyl acetate which was transferred to a GC vial containing pyridine and bis(trimethylsilyl) acetamide (Sigma Aldrich) and was then allowed to sit for at least 2 h at room temperature prior to GC analysis.

### Solid-state nuclear magnetic resonance analysis of polymers

High-resolution, solid-state ^13^C cross-polarization/magic angle spinning (CP/MAS) NMR spectra were collected at 4.7 T in a Bruker Avance 200 MHz spectrometer (50.13 MHz, room temperature). MAS was performed at 6900 Hz. A contact time of 2 ms was used with a sweep width of 21 kHz and 30,000 scans. The acquisition time was 0.024 s and the recycle delay was 2 s.

**Fig. 2:**
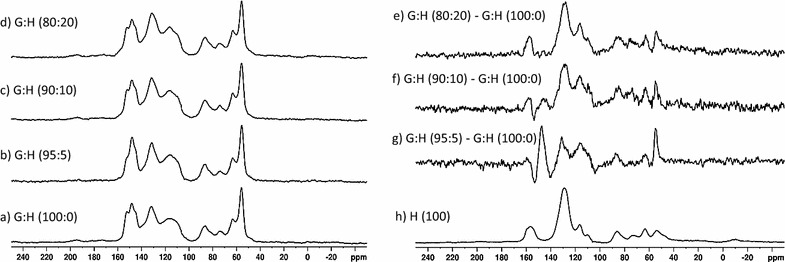
Solid-state NMR spectra: (a) G:H (100:0), (b) G:H (95:5), (c) G:H (90:10), (d) G:H (80:20). Subtraction spectra: (e) G:H (80:20)–G:H (100:0), (f) G:H (90:10)–G:H (100:0), (g) G:H (95:5)–G:H (100:0), and (h) H polymer spectrum

**Fig. 3:**
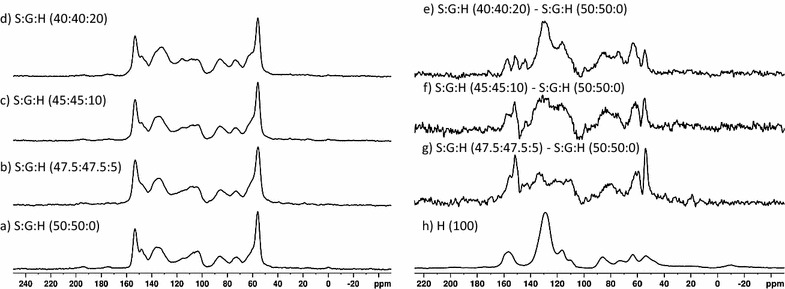
Solid-state NMR spectra: (a) S:G:H (50:50:0), (b) S:G:H (47.5:47.5:5), (c) S:G:H (45:45:10), (d) S:G:H (40:40:20). Subtraction spectra: (e) S:G:H (40:40:20)–S:G:H (50:50:0), (f) S:G:H (45:45:10)–S:G:H (50:50:0), (g) S:G:H (47.5:47.5:5)–S:G:H (50:50:0), and (h) H polymer spectrum

Difference spectra were generated to identify changes in the solid-state NMR spectra due to the addition of H into the reaction mixture. The spectrum was scaled until the intensity of the methoxyl peak at 56 ppm in the difference spectrum was at a null. This method assumes that the methoxyl content is not changing upon incorporation of H monomer, which is supported by the lack of methoxyl functional groups in H monomers. The resulting difference spectra are shown in Additional file 1: Figure S1 and Additional file 2: Figure S2. The intensities of the difference spectra shown in Figs. 2 and 3 were scaled to normalize the spectra to a consistent height and further highlight the differences between the polymers.

### Pyrolysis–MBMS analysis of lignin dehydrogenation polymers

Dehydrogenation polymers samples were pyrolyzed using a Frontier PY-2020 iD autosampler pyrolysis using He as a carrier gas. The pyrolysis vapors were analyzed using a VeraSpec MBx molecular beam mass spectrometer. The He flow rate was 0.9 L/min (STP) with a furnace temperature of 500 °C for a 1.5-min acquisition time per sample. The interface and transfer lines were maintained at 250 °C. The mass spectrometer ionization energy source was 17 eV.

## Results

### Lignin dehydrogenation polymer synthesis yields and thioacidolysis

Polymers synthesized readily from H and G monomers and solids were collected with yields (based on mass of starting material) around 70 wt%. The S DHP yield was 4% yield and the low amount of solid material collected did not allow for complete characterization. Inclusion of the H monomer in the S reaction mixture increased the yield to approximately 20% (characterization data provided in Additional file 5). The inability of S to polymerize has been reported previously and has been attributed to the formation of certain quinone methide compounds [14, 18–20]. S:G polymers were generated in yields of approximately 50% where the S:G:H (47.5:47.5:5) polymer recovery was lower (33%) due to inability to centrifuge all polymer into a pellet (some remained suspended in reaction media).

The amount of H-thioethylated products yielded from the H polymer, reported in Table 1, represented a small fraction (approximately 4%) of the polymer, indicating the H monomer did not form an abundance of linear, unbranched β-*O*-4 linkages.

**Table 1:** Thioacidolysis product yields and ratios as well as *M*_*w*_ as determined by GPC relative to polystyrene standards (standard deviations for duplicate thioacidolysis reactions)

Polymer	H (μmol/g sample)	S (μmol/g sample)	G (μmol/g sample)	Total recovery (μmol/g sample)	Mass % recovery (indicates % of β-*O*-4)	% H (in β-*O*-4, thioacidol.)	*M*_*w*_	*M*_*n*_	PD
H (100)	205 (± 11)	0 (± 0)	0 (± 0)	205 (± 11)	4	100 (± 0)	3600	1700	2.1
G (100:0)	0 (± 0)	0 (± 0)	404 (± 21)	404 (± 21)	10	0 (± 0)	7700	1400	5.6
S:G:H (50:50:0)	0 (± 0)	137 (± 9)	88 (± 8)	225 (± 17)	5	0 (± 0)	4800	1200	4.0
G (95:5)	60 (± 0)	0 (± 0)	465 (± 0)	525 (± 0)	11	11 (± 0)	2100	1600	1.4
G (90:10)	82 (± 6)	0 (± 0)	402 (± 31)	485 (± 36)	11	17 (± 0)	7500	1500	5.1
G (80:20)	118 (± 3)	0 (± 0)	346 (± 10)	464 (± 12)	11	25 (± 0)	7200	1500	4.9
S:G:H (47.5:47.5:5)	76 (± 2)	321 (± 16)	130 (± 22)	527 (± 40)	11	14 (± 1)	6500	1600	4.2
S:G:H (45:45:10)	160 (± 0)	362 (± 12)	380 (± 6)	902 (± 17)	20	18 (± 0)	5200	1300	4.0
S:G:H (40:40:20)	235 (± 8)	317 (± 21)	386 (± 20)	938 (± 49)	20	25 (± 0)	7800	1500	5.1

There was not a significant change in the amount of thioethylated products recovered from the G-based monomers upon incorporation of the H monomer into the starting reaction mixture, all being approximately 11%, similar to the recovery yield of the G:H (100:0) polymer. However, the H monomers recovered by thioacidolysis analysis was consistently higher than the H monomer concentration reacted during the polymer synthesis (Table 1) and the amount of G monomers recovered decreased with an increase in H content.

The S:G polymers showed a large increase in the amount of thioethylated products yielded upon incorporation of the H monomer into the polymer, increasing from 5 to 20 wt% of the original starting material [respectively, from S:G:H (50:50) to S:G:H (40:40:20)]. The increase in thioacidolysis product yield is attributed to the an approximate twofold increase in S monomers and an approximate fourfold increase in G monomers released in addition to the H monomers incorporated into the DHP polymers. Similar to what was observed in the thioacidolysis results of the G-based DHP polymers, the percentage of H measured by the thioacidolysis analysis of the S:G:H polymers was higher than the molar concentration of H monomers added to the synthesis reaction.

### Solid-state nuclear magnetic resonance analysis of polymers

Solid-state NMR spectra of H, and G:H polymers are shown in Fig. 2. Chemical shift assignments are based on previous work described in Kobayashi et al. [21] and are outlined in Table 2. H DHP lignin, shown in Fig. 2h, has a unique spectrum compared to the G:H (100:0) and S:G:H (50:50:0) spectra shown in Figs. 2a and 3a. Incorporation of H monomers into the G:H DHP polymers resulted in changes in the ratios of the peak intensities corresponding to the presence of etherified (152 ppm) and nonetherified structures (148 ppm) [21, 22]. A decrease in intensity of the shoulder at 152 ppm, and the corresponding relative increase in the 148 ppm peak in the G:H (95:5) spectrum (Fig. 2b), is attributed to a decrease in the etherified G, C3 carbons in the polymer relative to nonetherified structures [21]. Interestingly, the change in the ratio of etherified to nonetherified peaks in the NMR spectra becomes less prominent as the molar concentration of the H monomers in the reaction mixture was increased (Fig. 2c, d). Appearance of subtle shoulders at 131 and 156 ppm along with the new peak 117 ppm indicates the incorporation of H aromatic structures into the DHP lignins at higher starting H monomer concentration (Fig. 2c, d).

**Table 2:** Select solid-state ^13^C NMR chemical shift assignments [21, 22]

δ ^13^C (ppm)	Monomer origin	Assignment (carbon position, linkage)
156.2	H	C4
152.0	G	C3, C4 etherified
148.0	G	C3, C4 nonetherified
143.0	G	C_4_OH (free phenolic)
133.4	S. G	C1 (S or G) and/or C5 (G), 5–5
131	G	C1, 5–5 C4 nonetherified
128.9	H	C1, C2/C6, C3/C5 (R = aliphatic)
116.6	H	C3/C5, C4 etherified
116.0	G	C5
111.8	G	C2
110.6	H	C3/C5, C4 nonetherified
87.0	G	C_α_, β-5
86.4	H	C_α_, β-5
81.0	H	C_β_, β-*O*-4
73.9	H	C_α_, β-*O*-4
63.8	H	C_γ_, β-*O*-4
63.5	G	C_γ_, β-5
60.0	–	C_γ_, β-*O*-4
54.8	G	C_β_, β-5, β–β
54.5	H	C_β_, β-5, β–β
54.0	–	C_β_, β-5, β–β, also methoxyl

The difference spectra (Fig. 2e–g) highlight the small intensity changes observed in the ^13^C CP/MAS spectra. The difference spectrum generated by subtracting G:H (100:0) from G:H (95:5), shown in Fig. 2g, confirms the decrease in the etherified G3 carbons in the polymer relative to nonetherified structures as evidenced a negative peak at ~ 152 ppm and positive peak at ~ 148 ppm. There appears to be a negative peak at ~ 143 ppm that may be assigned to guaiacyl C_4_OH (free phenolic) in the G:H (100:0)–G:H (95:5) difference spectrum [21]. In addition, increases in the aromatic region between 110 and 150 ppm and the appearance of peaks at 54 ppm (C_β_ in β–β or β-5), 64 ppm (C_γ_ in β-5), and 87 ppm (C_α_ in β-5) are consistent with an increase in condensed units. As more H monomers were added to the reaction mixture, the difference spectrum began to show more clearly the inclusion of the H monomers into the polymer mixture (Fig. 2f and e). The spectrum difference generated from the subtraction of G:H (100:0) from G:H (90:10) (Fig. 2f) also shows a decrease in the etherified G C3 carbons in the polymer relative to nonetherified structures as evidenced a negative peak at ~ 152 ppm and positive peak at ~ 148 ppm. Interestingly, there is no evidence for a change in the ratio of the etherified to nonetherified structures in the G:H (100:0)–G:H (80:20) difference spectrum and the difference spectrum appears to be arising mainly from a primarily H-based polymer.

The ^13^C CP/MAS NMR spectra of the S:G:H polymers also show subtle changes in peak intensities as the H monomer concentration was increased in the starting reaction mixture (Fig. 3a–d). There are increases in peak intensity observed at ~ 59 ppm, the region 110–125, and 130 ppm. The changes observed in the ^13^C CP/MAS spectra peak intensities are more clearly highlighted in the difference spectra (Figs. 3e–g). The difference spectrum generated from the subtraction of the S:G:H (50:50:0) DHP polymer from the S:G:H (47.5:47.5:5) (Fig. 3g) shows an increase in the intensity of the shoulder at 152 ppm corresponding to an increase in the etherified units on G monomers relative to the S:G:H (50:50:0) DHP polymer along with an increase in intensity at ~ 143 ppm assigned to guaiacyl C_4_OH (free phenolic). In addition, there are increases in the aromatic region between 110 and 140 ppm that are assigned to G or H. The peak at 133 ppm is assigned to C5 in 5–5 structures or C1 in syringyl units. The difference spectrum generated from the subtraction the S:G:H (50:50:0) DHP polymer from the S:G:H (47.5:47.5:5) also shows increases in peaks at 54 ppm (C_β_ in β–β or β-5), 60 ppm (C_γ_ in β-*O*-4), and 81 ppm (C_β_ in β-*O*-4) consistent with the formation of more etherified linkages and the inclusion of H monomers into the DHP lignin framework (Fig. 3g). The difference spectrum generated from the subtraction of the S:G:H (50:50:0) DHP polymer from the S:G:H (45:45:10) shows a similar pattern to the S:G:H (50:50:0)–S:G:H (47.5:47.5:5) difference spectrum. There are increases observed in the aromatic region between 110 and 140 ppm (G or H aromatic ring carbons), at 60 ppm (C_γ_ in β-*O*-4), and 81 ppm (C_β_ in β-*O*-4), consistent again with the formation of more etherified linkages and the inclusion of H monomers into the DHP lignin framework in the S:G:H (45:45:10) DHP lignin. The difference spectrum of the S:G:H (50:50:0) polymer from the S:G:H (40:40:20) (Fig. 2e) shows incorporation of H aromatic units into the polymers as evidenced by the peaks between 60 and 90 ppm along with the peaks in the aromatic region at 116, 130, and 158 ppm (Fig. 2h). Resonances assigned to β-*O*-4 structures (60, 81 ppm) continue to increase relative to β–β structures (54 ppm). In addition, there are peaks observed at 152 ppm corresponding to an increase in the etherified units on G monomers along with an increase in intensity at ~ 143 ppm assigned to guaiacyl C_4_OH (free phenolic).

### Pyrolysis–MBMS analysis

**Fig. 4:**
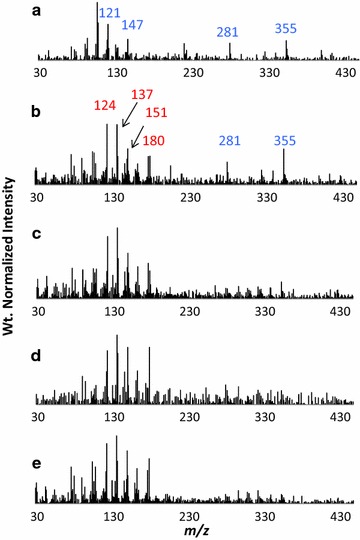
Py–MBMS spectra of lignin dehydrogenation polymers: **a** H, **b** G:H (80:20), **c** G:H (90:10), **d** G:H (95:5), **e** G:H (100:0). Blue corresponds to H-based ion fragments, red corresponds to G, and green corresponds to S-based ions

Pyrolysis of the lignin dehydrogenation polymers produced low molecular weight species assigned to H, G, and S monomers and dimers as shown in Table 3. Monomeric ions observed (< 210) were all distinctive of natural lignin monomers and their assignments have been given elsewhere [23–26]. As shown in Fig. 4a–e, the py–MBMS spectra of the H and G polymers both produce a spectral pattern that is unique to each polymer. The H polymer pyrolyzed to produce an abundance of ions *m/z* 281 and 355 assigned to H–H dimers (Fig. 4a).

**Table 3:** Origin of ions seen in py–MBMS spectra of lignin DHPs

	*m/z*	Pyrolysate assignment	Origin
Monomers	120	4-Vinylphenol	H
	121	–	H
	124	Guaiacol	G
	137	Coniferyl alcohol, homovanillin, 4-ethylguaiacol	G
	138	4-Methylguaiacol	G
	147	–	H
	150	4-Vinylguaiacol, p-coumaryl alcohol	G, H
	154	Syringol	S
	164	Allyl-, propenyl guaiacol	G
	167	Propiosyringone, syringylacetone, 4-ethylsyringol	S
	168	4-Methylsyringol, dimethoxyquinone	S
	178	Coniferyl aldehyde	G
	180	Coniferyl aldehyde, 4-vinylsyringol	G, S
	182	Syringaldehyde	S
	194	4-Propenylsyringol	S
	208	Sinapaldehyde	S
	210	Sinapyl alcohol	S
Dimers	281	–	H–H
	296	–	H–H
	298	–	H–H, G–G
	310	–	G–G
	316	–	H–H
	324	–	G–G
	341	–	G–G
	356	–	G–G
	358	–	G–G
	401	–	S–S
	418	–	S–S

Increases in H monomer concentration in the reaction mixture caused a decrease in the pyrolysis products assigned to G units in the G:H polymers relative to the G-based polymer, as evidenced by decreases in peak intensities assigned to G peaks in the weight normalized spectra (Fig. 4b–e). Incorporation of the H units into the G:H polymers is most clearly observed due to the appearance of peaks *m/z* 281 and 355 (H–H dimers) in G-based polymers upon incorporation of H into the starting reaction mixture (Fig. 4b and c).

**Fig. 5:**
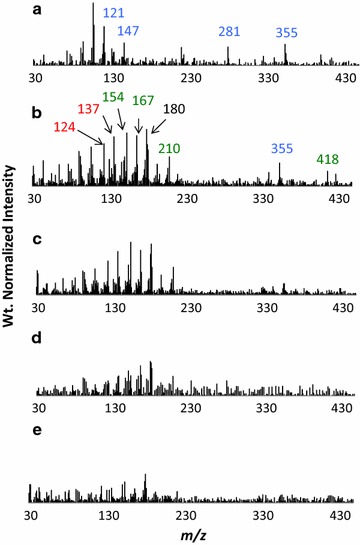
Py–MBMS spectra of lignin dehydrogenation polymers: **a** H, **b** S:G:H (40:40:20), **c** S:G:H (45:45:10), **d** S:G:H (47.5:47.5:5), **e** S:G:H (50:50:0). Blue corresponds to H-based ion fragments, red corresponds to G, and green corresponds to S-based ions

In contrast to what was observed in the G:H DHP lignins, the incorporation of the H monomer in S:G polymers increased the pyrolysis yields as evidenced by the increase in peak intensities associated with S and G units in the weight normalized pyrolysis spectra (Fig. 5b–e). In addition, the *m*/*z* 355 H–H dimer does not appear in the spectra until the S:G:H (40:40:20) DHP lignin (Fig. 5b) and there is little evidence for the presence of the *m*/*z* 281 H–H dimer in all spectra.

### Gel permeation chromatography analysis of lignin dehydrogenation polymers for average molecular weights

**Fig. 6:**
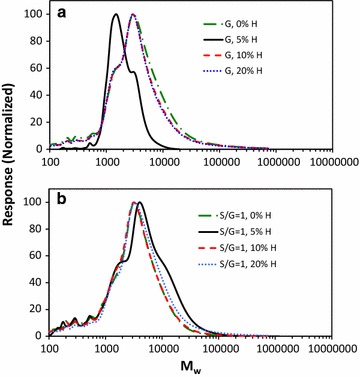
GPC chromatograms of acetylated lignin DHPs, **a** blue, G:H (80:20), red, G:H (90:10), black, G:H (95:5), green, G:H (100:0); **b** blue, S:G:H (40:40:20), red, S:G:H (45:45:10), black, S:G:H (47.5:47.5:5), green, S:G:H (50:50:0)

Gel permeation chromatography was used to determine the weight average molecular weight (*M*_*w*_), number average molecular weight (*M*_*n*_), and polydispersity (PD) of the polymers relative to polystyrene standards; the results reported in Table 1 and chromatograms are shown in Fig. 6. The H polymer had a low molecular weight at 3600 Da and the G polymer had the highest molecular weight of the homopolymers at 7700 Da.

Addition of 5% H monomers to the G monomer reaction mixture (G:H 95:5) lowered the *M*_*w*_ significantly from 7700 to 2100 Da. The GPC chromatogram of the G:H (95:5) DHP polymer (Fig. 6a) shows the decrease in *M*_*w*_ occurred due to lack of higher weight polymers (*M*_*w*_ > 10,000 Da) and an increase in polymers with lower molecular weights (~ 2000 Da). The molecular weight of the G:H polymers (G:H 90:10, G:H 80:20) began to approach the starting value of the G polymer (G:H 100:0) as the H monomer concentration increased. The GPC curves for the G:H (90:10) and G:H (80:20) DHP polymers (Fig. 6a) are similar to the G:H 100:0 DHP polymer with an increase in the higher molecular weight species (*M*_*w*_ > 10,000 Da) and a decrease in the lower molecular weight species weights (~ 2000 Da) relative to the G:H 95:5 DHP polymer.

Addition of 5% H monomers to the S:G monomer reaction mixture (S:G:H 47.5:47.5:5) increased *M*_*w*_ (6500 Da) relative to the S:G:H 50:50:0 polymer (4800 Da). Additionally, an increase in the shoulders appeared at ~ 1500 and ~ 15,000 Da in the GPC curve for the S:G:H (47.5:47.5:5) polymer (Fig. 6b). Similar to what was observed the addition of higher concentrations of H monomers to the G-based polymer (Table 1, Fig. 6a), the *M*_*w*_ for the S:G:H (45:45:10), and the S:G:H (40:40:20) DHP polymers were similar to the DHP polymer produced with no added H monomer S:G:H (50:50:0) (Table 1, Fig. 6b).

## Discussion

### Structural analysis of coumaryl alcohol (H) DHPs

The low molecular weight of the H DHP lignin relative to the other polymers is expected based on previous calculations and experimental studies [8, 27]. A low yield of thioethylated products from the pure H polymer may be due to an abundance of branching, likely from β–β (as observed in HSQC NMR spectra, data provided in Additional files 3, 4; Additional file 5: Tables S1, S2, S4) or 5–5 linkages which are enthalpically favorable for H-self coupling [9, 10, 28]. Branching has also been found by calculations and experimental evidence to be favorable during the polymerization of H monomers [9, 27, 28].

The presence of ions in the py–MBMS analysis at *m/z* 298, 296, and 281 have been detected from mass spectrometry analyses of H–H dimers previously [29] and are likely the result of fragmentation (during pyrolysis and/or ionization) of β–β and β-5 linked H dimers in the H polymer. The presence of *m/z* 355 in pure H lignin has not been described previously and is postulated here to be the result of the fragmentation of a trimer (C_27_H_28_O_6_ MW = 448) to form a phenol radical (C_6_H_5_O MW = 93) and a dimer radical fragment (C_21_H_23_O_5_ MW = 355) or from a trimer (C_27_H_26_O_6_ MW = 446) fragmenting to form a dimer radical fragment (C_21_H_23_O_5_ MW = 355) and radical fragment (C_3_H_7_O_3_ MW = 91).

### Alteration of G-based DHP structure occurs only at low concentrations of H monomers

The data discussed in the “Results” section indicates that at lower concentrations in the starting monomer solution, the H monomer alters the structure of the G-based polymers. A significant decrease in molecular weight was observed when H was incorporated into a G-based polymer synthesis at 5 mol% (Table 1, Fig. 6). The decrease in molecular weight accompanied by a decrease in etherified linkages and an increase in free phenolic structures indicates that the formation of G–H substrates did not support continued chain elongation leading to a decrease in the molecular weight of the polymer. Thioacidolysis and py–MBMS analyses show increases in condensed units based on subtle decreases in thioethylated monomer and pyrolysis product yields. The incorporation of H monomers directly into the G:H polymers led to an increase in the amount of condensed units and decrease in molecular weight supporting previous predictions that G–H and G–G β-5 “capping” substrates would inhibit chain elongation [8, 9].

At higher H monomer concentrations, the molecular weight of the G–H polymers increased and approached the molecular weight of the G:H (100:0) polymer indicating fewer capping substrates were forming. Smaller differences in the NMR spectra of the G:H (90:10, 80:20) polymers relative to the G:H (95:5) DHP spectra were observed (difference spectra shown to scale in Additional file 1: Figure S1). The clearest evidence for the absence of reactivity between H and G monomers is observed in the G:H (80:20)–(100:0) difference spectrum (Fig. 2e) in which only an H–H homopolymer is observed and there appears to be little to no observable differences in the G–G polymer relative to the G-only polymer (Fig. 2d). The presence of H–H homopolymer in the G:H (80:20) polymer, and to a smaller extent, the G:H (90:10) polymer, is also observed in the py–MBMS spectra (Fig. 4) based on the presence of the ions at *m/z* 281 and 355 (H–H dimers). The NMR difference spectra and py–MBMS spectra support DFT calculations that predict H monomers would preferentially react with other H monomers, creating H-based homopolymers in the presence of the mixture of G:H polymers and G homopolymers [8, 9].

### Structural changes in S:G-based polymers upon incorporation of H monomers

Addition of H monomers into the S:G reaction mixture had a greater influences on the structure of the S:G-based polymer in comparison to the G-based polymer (difference spectra shown to scale in Additional file 2: Figure S2). Consistent with the addition of H monomers to the G monomer DHP synthesis, the initial addition of H to the S:G reaction mixture inhibited chain elongation due to the formation of G–H capping structures as observed by the presence of the lower molecular weight shoulders in the GPC measurements for the S:G:H (47.5:47.5:5) DHP lignins. However, in contrast to the G:H (95:5) polymer, a higher molecular weight fraction was observed in the S:G:H (47.5:47.5:5) polymer presumably due to a lower probability of forming G–G β-5 capping structures because of the lower G monomer concentration in the reaction mixture and the S monomer presence [8, 27]. Interestingly, the S:G:H (40:40:20) formed the highest molecular weight polymer even though the concentration of H monomers was the highest of the starting reaction mixtures. We attribute the higher molecular weight to H monomers preferentially reacting with other H monomers rather than with either S or G substrates as evidenced by the observation of an H homopolymer in both the solid-state NMR and py–MBMS spectra (Figs. 3 and 5) thus lowering the probability of both G–H and G–G β-5 “capping” reactions. Consistent with previous theoretical calculations, the formation of H homopolymers at higher concentrations of H monomer is observed in the G and S:G-based polymers [8, 9].

The solid-state NMR difference spectrum indicates an increase in the formation of 5–5 bonds between aromatic units indicating that reactions forming 5–5 linkages between H monomers are occurring similar to what was previously observed by Syrjänen and Brunow [28]. We also observed an increase in β-*O*-4 linkages indicating that H and S must preferentially react to form these types of structures. The preference to form β-*O*-4 linkages between S and H monomers supported by higher yields of both H and S products by thioacidolysis (Additional file 5: Table S3) and previous reports of the higher reactivity of S monomers in the presence of H monomers [14, 18–20, 30]. Additionally, lower molecular weights would be observed if an abundance of G–H substrates were forming, also indicating that H must be reacting with S. Thioacidolysis and py–MBMS analyses also show increased recoveries of G monomers in linear ether linkages relative to S monomers. However, the overall S/G ratio did not appear to change substantially in the solid-state NMR spectra of the S:G:H polymers (Fig. 3a–d) indicating a preference of S and H monomers to react together, rather than an increase in ether linkages due solely to an increase in S monomer content. The solid-state ^13^C CPMAS S:G:H (40:40:20) and S:G:H (45:45:10) difference spectra also indicate increases in the abundance of free phenolic substrates relative to the S:G:H (50:50:0) and S:G:H (47.5:47.5:5) DHP lignins. A rise in the number of free phenolic groups along with a simultaneous increase in β-*O*-4 linkages indicates a potential increase in the amount of branching present in the polymer mixture.

## Conclusions

A Zutropfverfahren method was used to polymerize lignin dehydrogenation polymers from coniferyl, sinapyl, and coumaryl alcohols with horseradish peroxidase and hydrogen peroxide containing varying S:G:H compositions. The Zutropf synthesis method provided a reasonable experimental validation for theoretical calculations of lignin monomers intrinsic reactivity and how they influence the structure of the resulting polymer. Previously, it was predicted that the incorporation of H monomers into lignin could result in lower molecular weight polymers [8] and that H monomers were more likely to self-couple than couple with other monomers [9]. Our studies indicate that H would influence the molecular weights of the polymers but it depends on the relative abundance and type of other monomers present. For example, in agreement with hypotheses based on DFT calculations [8, 9], low concentrations of H monomer incorporation on G-based polymers result in a decrease in molecular weight. It also appears that H monomers do indeed exhibit preferential binding to other H monomers, forming what appears to be clusters of H-based polymer even in the presence of S and/or G monomers when the abundance of H monomers approaches 20 mol%.

The increase in H content leads to an increase in molecular weight of S:G polymers and also an increase in linear ether linkages. The changes in the lignin dehydrogenation polymers structures and molecular weights upon incorporation of the H monomer support previous findings indicating that high-H lignin in biomass likely contains separate H and S:G lignin polymers [4]. Overall, the addition of the H caused structural changes that either allowed for a greater release of monomeric species by thermal and chemical deconstruction due to more labile ether linkages or reduced the size of the lignin polymers. A decrease in molecular weight of G-based polymers or an increase in linear β-*O*-4 or other ether linkages in S:G-based polymers should influence the recalcitrant nature of biomass. Our results show that the incorporation of low concentrations of H monomers during lignin polymerization may help make biomass less recalcitrant and more amenable to processing for biorefinery applications [31, 32].

## Additional files

**Additional file 1: Figure S1.** Solid-state CP/MAS NMR spectra: a) G:H (100:0), b) G:H (95:5), c) G:H (90:10), d) G:H (80:20). Subtraction spectra: e) G:H (100:0), f) G:H (95:5)–(G:H 100:0), g) G:H (90:10)–G:H (100:0), and h) G:H (80:20)–G:H (100:0). Spectra are all scaled to the same intensity. Subtraction spectra are between 14 and 18% of the intensity of the G:H (100:0) spectrum.

**Additional file 2: Figure S2.** Solid-state CP/MAS NMR spectra: a) S:G:H (50:50:0), b) S:G:H (47.5:47.5:5), c) S:G:H (45:45:10), d) S:G:H (40:40:20). Subtraction spectra: e) G:H (100:0), f) S:G:H (47.5:47.5:5)–S:G:H (50:50:0), g) S:G:H (45:45:10)–S:G:H (50:50:0), and h) S:G:H (40:40:20)–S:G:H (50:50:0). Spectra are all scaled to the same intensity. Subtraction spectra are between 14 and 22% of the intensity of the S:G:H (50:50:0) spectrum.

**Additional file 3: Figure S3.** HSQC NMR spectra showing bond regions and aromatic regions of lignin dehydrogenation polymers from G/H monomers.

**Additional file 4: Figure S4.** HSQC NMR spectra showing bond regions and aromatic regions of select lignin dehydrogenation polymers from S, G and/or H monomers.

**Additional file 5.** Supplementary Methods, Tables, and Figure Captions.

## Abbreviations

CP/MAS: cross polarization magic angle spinning
DFT: density functional theory
DHP: dehydrogenation polymer
GPC: gel permeation chromatography
NMR: nuclear magnetic resonance
Py–MBMS: pyrolysis–molecular beam mass spectrometry
Zutropf: Zutropfverfahren
