# Supplementary figures and images for: The effect of coumaryl alcohol incorporation on the structure and composition of lignin dehydrogenation polymers
Source: Biotechnol Biofuels. 2017 Nov 30;10:281. doi: 10.1186/s13068-017-0962-2 (PMC5707875; doi:10.1186/s13068-017-0962-2)

## Slide 1
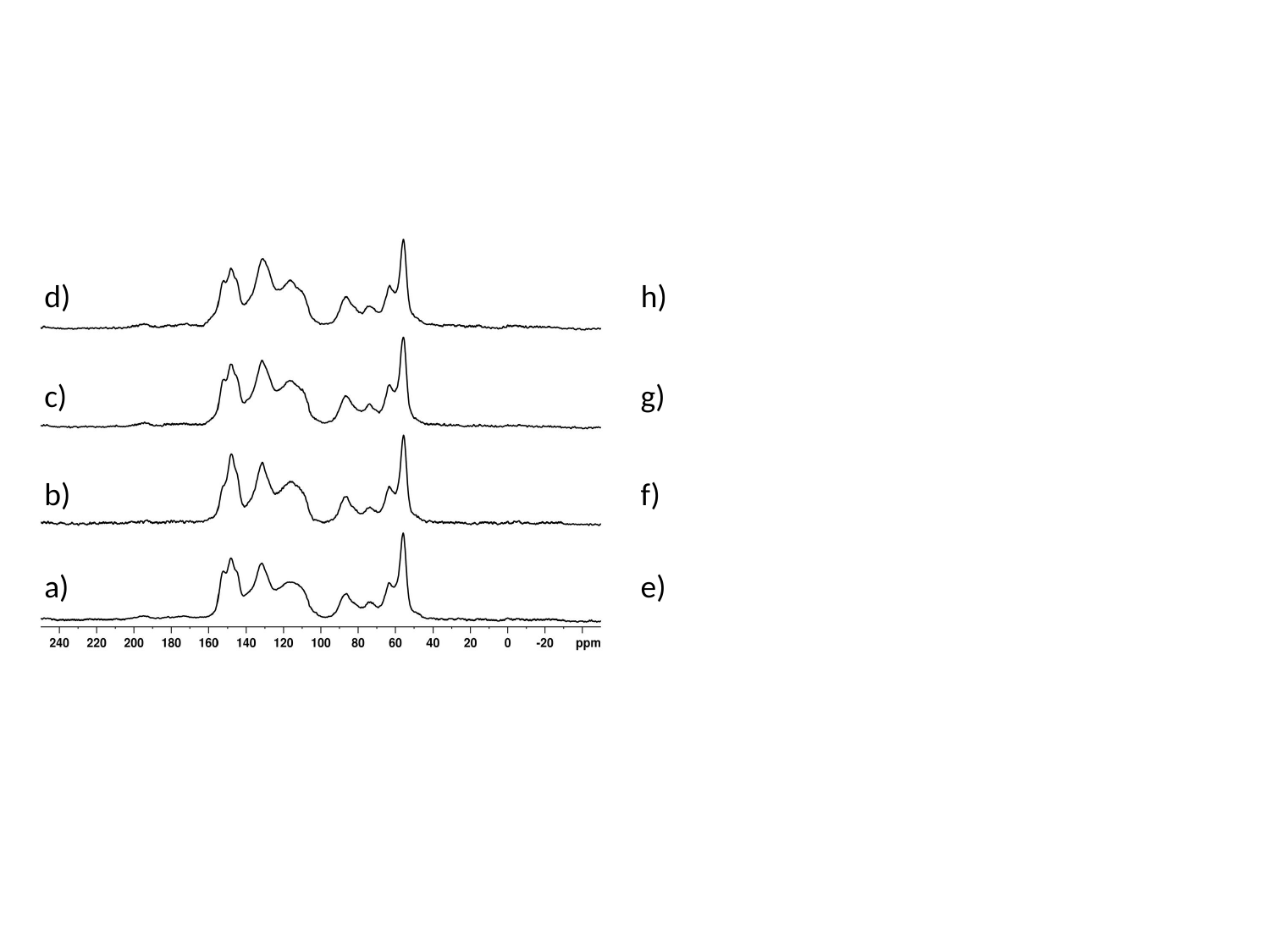

d)
h)
c)
g)
b)
f)
a)
e)

Supplement: Supplementary file 1 — Additional file 1: Figure S1. Solid-state CP/MAS NMR spectra: a) G:H (100:0), b) G:H (95:5), c) G:H (90:10), d) G:H (80:20). Subtraction spectra: e) G:H (100:0), f) G:H (95:5)–(G:H 100:0), g) G:H (90:10)–G:H (100:0), and h) G:H (80:20)–G:H (100:0). Spectra are all scaled to the same intensity. Subtraction spectra are between 14 and 18% of the intensity of the G:H (100:0) spectrum. [file 13068_2017_962_MOESM1_ESM.pptx]

## Slide 1
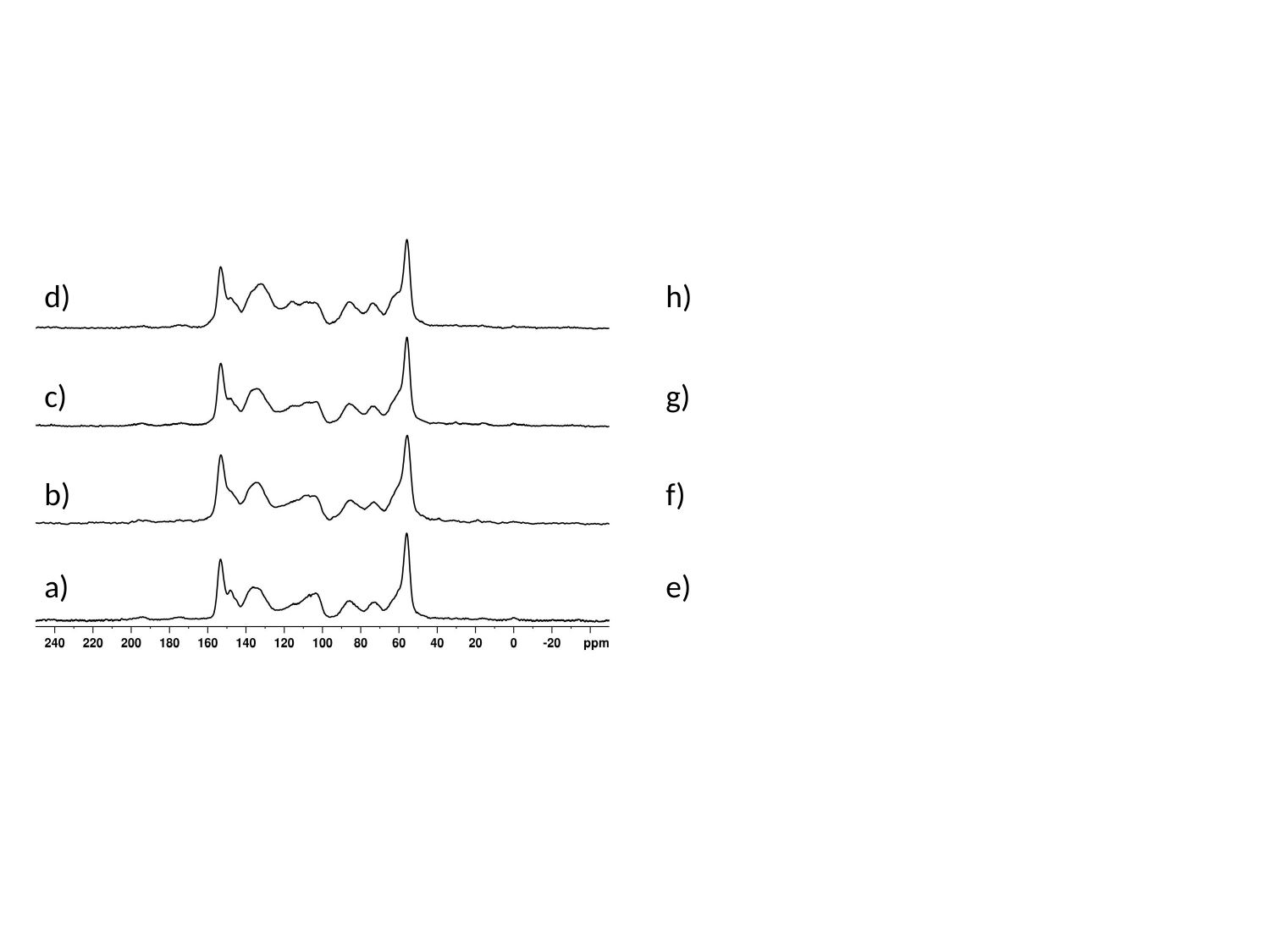

d)
h)
c)
g)
b)
f)
a)
e)

Supplement: Supplementary file 2 — Additional file 2: Figure S2. Solid-state CP/MAS NMR spectra: a) S:G:H (50:50:0), b) S:G:H (47.5:47.5:5), c) S:G:H (45:45:10), d) S:G:H (40:40:20). Subtraction spectra: e) G:H (100:0), f) S:G:H (47.5:47.5:5)–S:G:H (50:50:0), g) S:G:H (45:45:10)–S:G:H (50:50:0), and h) S:G:H (40:40:20)–S:G:H (50:50:0). Spectra are all scaled to the same intensity. Subtraction spectra are between 14 and 22% of the intensity of the S:G:H (50:50:0) spectrum. [file 13068_2017_962_MOESM2_ESM.pptx]
