# Supplementary material for: The effect of coumaryl alcohol incorporation on the structure and composition of lignin dehydrogenation polymers
Source: Biotechnol Biofuels. 2017 Nov 30;10:281. doi: 10.1186/s13068-017-0962-2 (PMC5707875; doi:10.1186/s13068-017-0962-2)
Supplement: Supplementary file 3 — Additional file 3: Figure S3. HSQC NMR spectra showing bond regions and aromatic regions of lignin dehydrogenation polymers from G/H monomers. [file 13068_2017_962_MOESM3_ESM.pptx]

## Slide 1
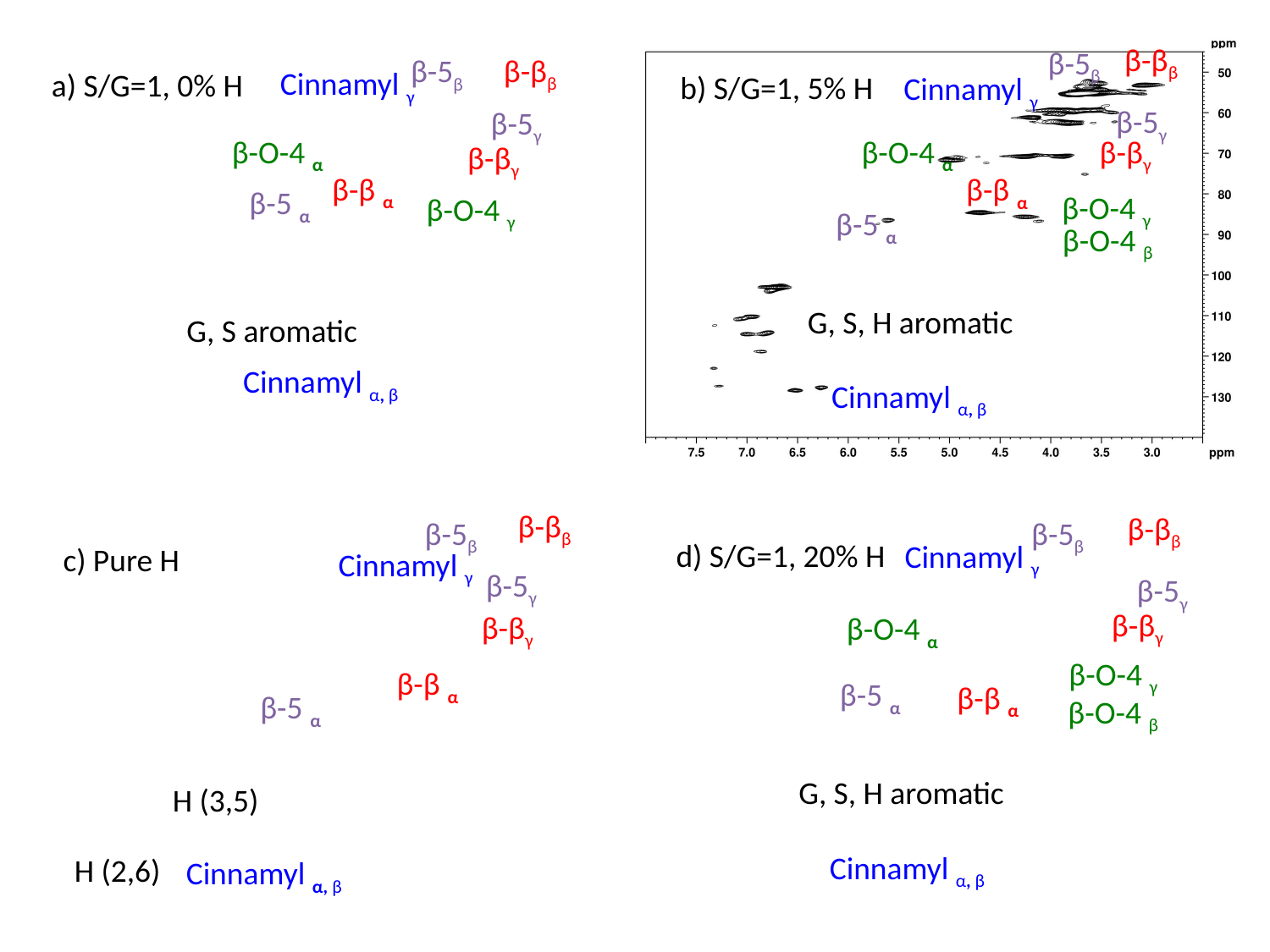

β-ββ
β-5β
β-ββ
β-5β
Cinnamyl γ
a) S/G=1, 0% H
b) S/G=1, 5% H
Cinnamyl γ
β-5γ
β-5γ
β-O-4 α
β-O-4 α
β-βγ
β-βγ
β-β α
β-β α
β-5 α
β-O-4 γ
β-O-4 γ
β-5 α
β-O-4 β
G, S, H aromatic
G, S aromatic
Cinnamyl α, β
Cinnamyl α, β
β-ββ
β-5β
c) Pure H
Cinnamyl γ
β-5γ
β-βγ
β-β α
β-5 α
Cinnamyl α, β
H (3,5)
H (2,6)
β-ββ
β-5β
d) S/G=1, 20% H
Cinnamyl γ
β-5γ
β-βγ
β-O-4 α
β-O-4 γ
β-5 α
β-β α
β-O-4 β
G, S, H aromatic
Cinnamyl α, β
