# Supplementary material for: The effect of coumaryl alcohol incorporation on the structure and composition of lignin dehydrogenation polymers
Source: Biotechnol Biofuels. 2017 Nov 30;10:281. doi: 10.1186/s13068-017-0962-2 (PMC5707875; doi:10.1186/s13068-017-0962-2)
Supplement: Supplementary file 4 — Additional file 4: Figure S4. HSQC NMR spectra showing bond regions and aromatic regions of select lignin dehydrogenation polymers from S, G and/or H monomers. [file 13068_2017_962_MOESM4_ESM.pptx]

## Slide 1
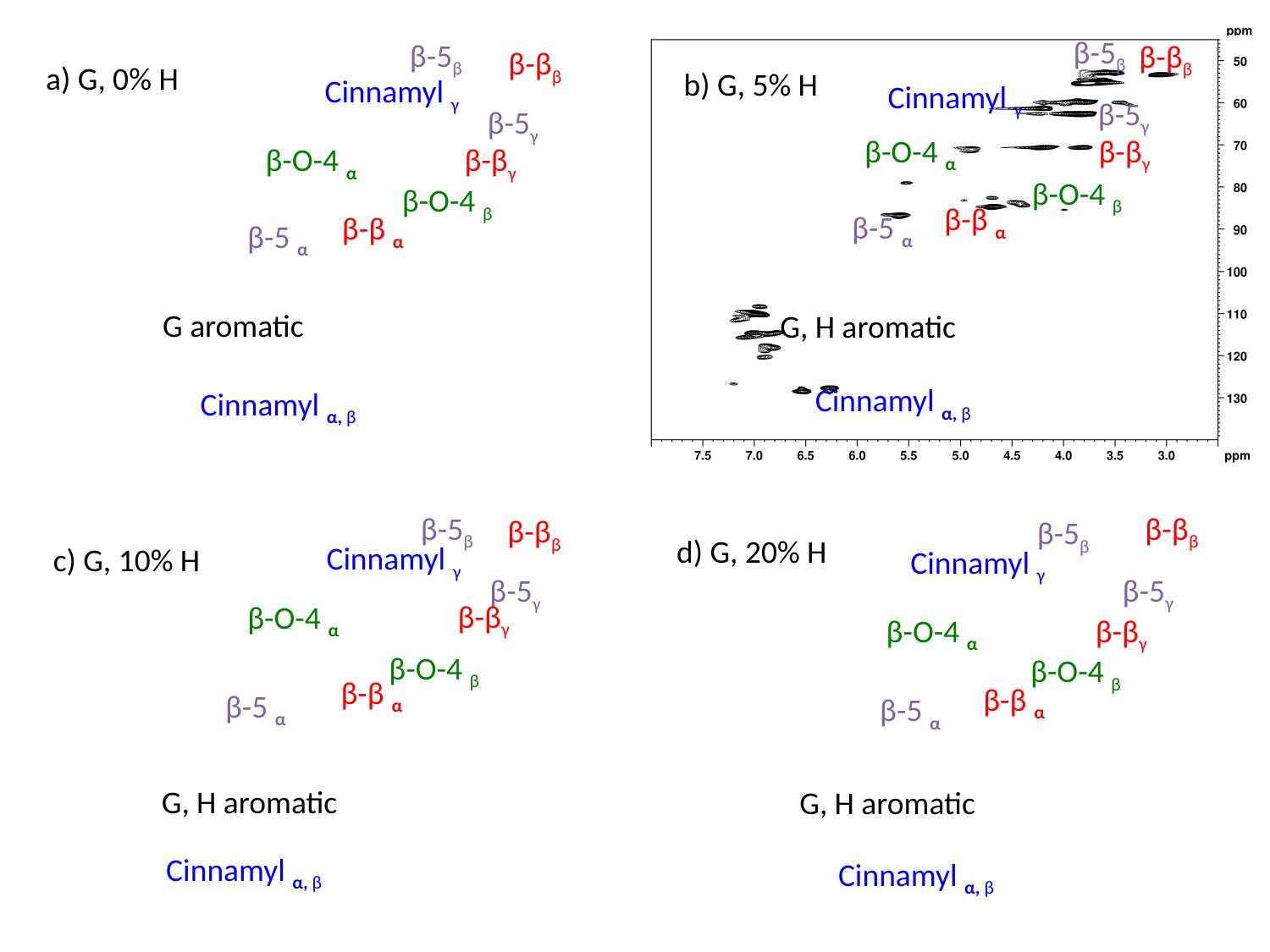

b) G, 5% H
β-5β
β-ββ
Cinnamyl γ
β-5γ
β-O-4 α
β-βγ
β-O-4 β
β-β α
β-5 α
G, H aromatic
Cinnamyl α, β
β-5β
β-ββ
a) G, 0% H
Cinnamyl γ
β-5γ
β-O-4 α
β-βγ
β-O-4 β
β-β α
β-5 α
G aromatic
Cinnamyl α, β
β-5β
β-ββ
Cinnamyl γ
β-5γ
β-βγ
β-O-4 α
β-O-4 β
β-β α
β-5 α
G, H aromatic
Cinnamyl α, β
c) G, 10% H
β-ββ
β-5β
Cinnamyl γ
β-5γ
β-βγ
β-O-4 β
β-β α
β-5 α
G, H aromatic
Cinnamyl α, β
d) G, 20% H
β-O-4 α
