# Supplementary material for: The effect of coumaryl alcohol incorporation on the structure and composition of lignin dehydrogenation polymers
Source: Biotechnol Biofuels. 2017 Nov 30;10:281. doi: 10.1186/s13068-017-0962-2 (PMC5707875; doi:10.1186/s13068-017-0962-2)
Supplement: Supplementary file 5 — Additional file 5. Supplementary Methods, Tables, and Figure Captions. [file 13068_2017_962_MOESM5_ESM.docx]

**Additional Methods**

**Liquid-State NMR Analysis of Lignin DHPs**

2D ^1^H-^13^C HSQC NMR experiments were conducted on a 600 MHz Bruker Avance III (14.7 T) spectrometer. 15-40 mg of polymers were solubilized in 400 μL 4:1 (v:v) DMSO-d_6_:pyridine-d_5_ and analyzed using a modified procedure similar to that reported in Mansfield et al. [1]. The polymers were sonicated and vortexed in an effort to solubilize and produce a homogenous solution (likely oligomers), although some of the polymer settled to the bottom of the NMR tubes. There is uncertainty associated with the representation of the polymers provided by the HSQC spectra due to the lack of polymer solubility in the solvent. HSQC parameters were as follows: 256 scans, 1024 transients in F2 and 400 transients in F1, 0.5 s delay, F1 acquisition time of 0.006 s and F2 acquisition time of 0.07 s. The F2 sweep width was 12 ppm and F1 was 220 ppm. Experiments and integral values were performed at 40°C (S-containing polymers at 75°C improved spectral data quality) and data were processed with Guassian apodization in F2, cosine bell apodization in F1 and zero filled to a final matrix size of 2 k by 1 k. ^13^C NMR spectra of DHP lignin samples were also acquired on a 600 MHz Bruker Avance III (14.7 T) spectrometer using a room temperature BBO probe. Spectra were collected using a 90° pulse with an inverse-gated decoupling pulse sequence, a 10 second pulse delay, and 14k scans at 40°C. 15-40 mg of polymers were dissolved in 4:1 DMSO-d_6_:Pyridine-d_5_.

Table S1. HSQC NMR spectra integration regions and identities.

| Structural identity (position) | ^1^H (ppm) | ^13^C (ppm) |
| --- | --- | --- |
| β-O-4 (α) | 4.99 | 71.45 |
| β-O-4 (β, S) | 4.24 | 85.35 |
| β-O-4 (β, G/H) | 4.44 | 83.63 |
| β-O-4 (γ, S) | 4.31 | 61.45 |
| β-5 (α) | 5.60 | 86.63 |
| β-5 (β) | 3.58 | 52.81 |
| β-5 (γ) | 3.85 | 62.5 |
| β- β (α) | 4.71 | 84.71 |
| β- β (β) | 2.95 | 53.34 |
| β- β (γ) | 4.28, 3.85 | 70.80, 70.60 |
| Cinnamyl end group (α) | 6.52 | 128.30 |
| Cinnamyl end group (β) | 6.26 | 127.90 |
| Cinnamyl end group (γ) | 3.95 | 59.28 |
| S (2,6) | 6.72 | 103.22 |
| G (2) | 7.00 | 110.32 |
| G (3,5) | 6.98 | 114.32 |
| G (3) | 6.79 | 113.90 |
| G (5 ether) | 7.07 | 115.56 |
| G (6) | 6.86 | 118.60 |
| G/H (3,5) | 6.82 | 115.01 |
| H (2,6) | 7.23 | 126.95 |
| H (3,5) | 6.95 | 115.80 |

Table S2. Relative percentages of bond types seen in liquid-state HSQC NMR spectra of lignin dehydrogenation polymers.

| Polymer | % β-O-4 | % β-5 | % β-β | % Cinnamyl end | S/G (NMR) | % H (NMR)* |
| --- | --- | --- | --- | --- | --- | --- |
| H (100) | 19.2 | 24.6 | 37.0 | 19.2 | - | - |
| G (100:0) | 21.1 | 31.9 | 22.9 | 24.1 | - | - |
| G (95:5) | 22.1 | 28.0 | 26.9 | 23.0 | - | 1.5 |
| G (90:10) | 23.2 | 24.6 | 23.5 | 28.7 | - | 12 |
| G (80:20) | 21.1 | 28.1 | 24.2 | 26.6 | - | 19 |
| S:G:H (50:50:0) | 18.6 | 23.0 | 34.0 | 24.4 | 1.45 | - |
| S:G:H (47.5:47.5:5) | 24.4 | 20.3 | 27.5 | 27.9 | 1.29 | 3.6 |
| S:G:H (45:45:10) | 22.6 | 20.1 | 31.7 | 25.6 | 1.32 | 12 |
| S:G:H (40:40:20) | 22.5 | 21.7 | 27.7 | 28.1 | 0.92 | 20 |

*H% = 100*Vol H(2/6)/ [Vol H(2/6) + Vol S(2/6) + 2*(Vol G2)]

Table S3: Thioacidolysis and GPC data from S-based polymers.

| Polymer | Polymer yield | H (μmol/g sample) | S (μmol/g sample) | G (μmol/g sample) | Total recovery (μmol/g sample) | S/G (thioacidol.) | % H (in β-O-4, thioacidol.) | Mw | Mn | PD |
| --- | --- | --- | --- | --- | --- | --- | --- | --- | --- | --- |
| S (100:0) | 4% | 0 | 94 | 0 | 94 | - | 0.0 | 1600 | 520 | 3.0 |
| S (90:10) | 20% | 131 (± 1) | 393 (± 3) | 0 | 524 (± 4) | - | 25 (± 0) | 7400 | 1100 | 6.5 |
| S (80:20) | 20% | 474 (± 51) | 535 (± 49) | 0 | 1010 (± 99) | - | 47 (± 0) | 4000 | 1300 | 3.0 |

Table S4. Relative percentages of bond types seen in liquid-state HSQC NMR spectra of S-based lignin dehydrogenation polymers.

| Polymer | % β-O-4 | % β-5 | % β-β | % Cinnamyl end | S/G (NMR) | % H (NMR) |
| --- | --- | --- | --- | --- | --- | --- |
| S (100:0) | - | - | - | - | - | - |
| S (90:10) | 17.2 | 19.3 | 31.2 | 32.4 | - | 13 |
| S (80:20) | 18.9 | 6.9 | 44.2 | 29.9 | - | 25 |

References

1. Mansfield, S.D., et al., *Whole plant cell wall characterization using solution-state 2D NMR.* Nature Protocols, 2012. **7**(9): p. 1579-1589.
